# Quo vadis Radiomics? Bibliometric analysis of 10-year Radiomics journey

**DOI:** 10.1007/s00330-023-09645-6

**Published:** 2023-04-18

**Authors:** Stefania Volpe, Federico Mastroleo, Marco Krengli, Barbara Alicja Jereczek-Fossa

**Affiliations:** 1 Division of Radiation Oncology, IEO European Institute of Oncology, IRCCS, Via Ripamonti 435, 20141 Milan, Italy; 2 Department of Oncology and Hemato-Oncology, University of Milan, Via Festa del Perdono 7, 20122 Milan, Italy; 3 Department of Translational Medicine, University of Piemonte Orientale, Via Solaroli 17, 28100 Novara, Italy; 4 Division of Radiation Oncology, University Hospital “Maggiore Della Carità”, Corso Mazzini 18, 28100 Novara, Italy

**Keywords:** Diagnostic imaging, Bibliometrics, Machine learning

## Abstract

**Objectives:**

Radiomics is the high-throughput extraction of mineable and—possibly—reproducible quantitative imaging features from medical imaging. The aim of this work is to perform an unbiased bibliometric analysis on Radiomics 10 years after the first work became available, to highlight its status, pitfalls, and growing interest.

**Methods:**

Scopus database was used to investigate all the available English manuscripts about Radiomics. R Bibliometrix package was used for data analysis: a cumulative analysis of document categories, authors affiliations, country scientific collaborations, institution collaboration networks, keyword analysis, comprehensive of co-occurrence network, thematic map analysis, and 2021 sub-analysis of trend topics was performed.

**Results:**

A total of 5623 articles and 16,833 authors from 908 different sources have been identified. The first available document was published in March 2012, while the most recent included was released on the 31st of December 2021. China and USA were the most productive countries. Co-occurrence network analysis identified five words clusters based on top 50 authors’ keywords: Radiomics, computed tomography, radiogenomics, deep learning, tomography. Trend topics analysis for 2021 showed an increased interest in artificial intelligence (*n* = 286), nomogram (*n* = 166), hepatocellular carcinoma (*n* = 125), COVID-19 (*n* = 63), and X-ray computed (*n* = 60).

**Conclusions:**

Our work demonstrates the importance of bibliometrics in aggregating information that otherwise would not be available in a granular analysis, detecting unknown patterns in Radiomics publications, while highlighting potential developments to ensure knowledge dissemination in the field and its future real-life applications in the clinical practice.

**Clinical relevance statement:**

This work aims to shed light on the state of the art in radiomics, which offers numerous tangible and intangible benefits, and to encourage its integration in the contemporary clinical practice for more precise imaging analysis.

**Key Points:**

• *ML-based bibliometric analysis is fundamental to detect unknown pattern of data in Radiomics publications.*

• *A raising interest in the field, the most relevant collaborations, keywords co-occurrence network, and trending topics have been investigated*.

• *Some pitfalls still exist, including the scarce standardization and the relative lack of homogeneity across studies*.

**Supplementary Information:**

The online version contains supplementary material available at 10.1007/s00330-023-09645-6.

## Introduction

Radiomics is an innovative and emerging field that focuses on the high-throughput extraction of mineable and—possibly—reproducible quantitative imaging features from routinely acquired medical imaging. Radiomics is based on the hypothesis that a quantitative analysis of medical images by specific software can provide the physician with more information that otherwise would have not been possible to infer. The radiomic analysis starts with the acquisition of high quality and standardized imaging, in which a region of interest is manually or automatically delineated. Quantitative imaging features, which rely on the delineated region and surroundings, are extracted from the defined area and analyzed. After a process of features selection, the most informative features are identified even relying on their independence from other sources, which could potentially include clinical, genomic, proteomic data.

Results from the groundbreaking publication by Lambin et al. in 2012 [1] have laid the basis for the main rationale for Radiomics studies, which is the identification of image-based biomarkers for diagnostic, prognostic, or predictive purposes. To date, several studies have been published on the correlation between radiomic features and specific disease phenotypes (e.g., benign vs malignant lesions [2, 3]), genotypes (e.g., lung [4] and gynecological cancers [5]), and treatment response (e.g., head and neck cancers [6]). Given these premises, the cost-effectiveness of Radiomics studies and the broad availability of digitalized imaging, it is quite straightforward to understand why Radiomics have rapidly become a trend topic in the field of Oncology.

While several narrative and systematic reviews, and some metanalyses have been published, the topic of these works has been necessarily specialistic, with dedicated focus on specific diseases, imaging modalities, and/or methodological aspects [7–10].

We could state that, even if a large amount of published research is present, outside of academic literature there is still a limited range of clinical application of these technologies, which, in addition, have not been easily translated as commercial products [11]. In addition, the continuous development of new data tools may potentially lead to a hazardous delay in the clinical implementations, as originally intended, leading to a mismatch between the need of more consolidated literature in clinical practice and the daily availability of new sophisticated and promising tools which have not been tested as bedside allies. In this fragmented scenario, recent studies have been providing new scores to assess the quality of science and reporting of radiomics in oncologic studies, resulting in a need of further literature consolidation and a deeper analysis of current literature.

Bibliometrics is a rigorous methodology for the analysis of large quantity of literature data and metadata, coming from high-quality public available scientific databases. This big-data methodology is easily accessible, reproducible, and objective, not including the human interaction step in the qualitative evaluation of the analyzed manuscripts such as it happens in other forms of literature analysis. Furthermore, it helps to highlight the evolutionary steps undertaken by a specific field, while revealing promising areas and future developments [12, 13].

The aim of this work is to perform an unbiased bibliometric analysis on Radiomics 10 years after the first work on this topic became available to the scientific community and to analyze how the scientific interest and the harmonization in this field is growing. Collaboration networks, trending keywords, citation analysis, and thematic maps will have been built and analyzed to provide a comprehensive overview on Radiomics research, to underline its strengths and weaknesses, and to critically orient future publications in the field.

## Materials and methods

### Data origin and search strategy

Scopus electronic documents database was used as data origin for the executed analysis. The search strategy was set per the following criteria: “Radiomics” has been the only term investigated in title, abstract, and keywords, and all types of documents were accepted. Only works in English were considered eligible. Timespan of considered documents included the first published document in 2012 up to the last published document in 2021. The list of available documents has been exported together with the corresponding metadata on the 9th of April 2022. The search was conducted per the following string: “TITLE-ABS-KEY (Radiomics) AND (LIMIT-TO (LANGUAGE, "English")) AND (EXCLUDE (PUBYEAR, 2022))”. A workflow diagram is available as supplementary material.

### Data preprocessing

Data, comprehensive of full set of metadata, were exported from Scopus using BibTex file format. R code (R version 4.2.0) with Bibliometrix package was used for data analysis [14].

BibTex data files were imported in R and then converted in multiple dataframes (command used: convert2df). The obtained dataframes were subsequently grouped to a single dataframe, which has been used as data source for the analysis.

### Data analysis

The initial interpretation of the data was performed by the “biblioAnalysis()” command and “summary()” functions from Bibliometrix package. This includes the following information: time distribution of the articles, number of documents and types, average years from publications, average citations per documents, average citations per documents, number of authors, number of single and multi-authored documents, number of co-authors per document, most productive authors, top manuscripts per citations, corresponding author’s countries and number of single/multiple country publications, total citations per country, most relevant sources, most relevant keywords. Plots of the main characteristics were realized.

Collaboration networks were investigated by “metaTagExtraction” and “Biblionetwork” commands, and “Networkplot” was then used for graphical representation.

“Biblioshiny()” command was also used to perform the following analysis: country scientific collaborations, institution collaboration networks, keyword analysis, comprehensive of co-occurrence network, thematic map analysis, and 2021 trend topics focus.

Quantitative text analysis in document titles has been performed using “dplyr”, “stringr”, “tidytext”, and “ggplot2” R libraries.

## Results

### Dataset

**Fig. 1:**
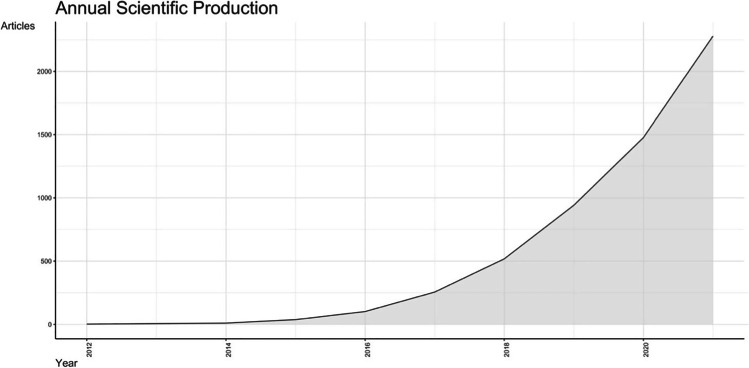
Annual scientific production chart from 2012 to 2021

A total of 5623 articles was identified. The first available document was published in March 2012 [1], while the most recent was released on the 31st of December 2021 [15]. In 2012, two articles were present, and the increasing interest in the topic leads to annual growth rate of publication equal to 118.6% (Fig. 1), thus reaching a number of 2278 articles published in year 2021.

The most represented document type is scientific article (68.9%, *n* = 3829), followed by review (13.5%, *n* = 758) and conference paper (10.7%, *n* = 604). Books and book chapters represented the 0.03% and the 0.8% of the total scientific output (*n* = 2 and 45, respectively).

### Sources

A total of 908 different sources of documents have been identified. Considering the scientific journals, the most represented was “Frontiers in Oncology” (409 articles, 7.3%, IF 6.24), followed by “European Radiology” (255 articles, 4.5%, IF 5.31), “Scientific Reports” (208 articles, 3.7%, IF 4.37), “Progress in Biomedical Optics and Imaging—Proceedings of SPIE” (201 articles, 3.6%, IS 0.67), and “Cancers” (154 articles, 2.7%, IF 6.64).

### Authors and collaborations

Overall, 16,833 different authors were comprised in the analyzed documents. An average number of 7.89 co-authors per document have been esteemed and 239 single-authored docs were found.

The five most productive authors are Wang Y, 191 documents; Tian J, 179 documents; Zhang Y, 144 documents; Li Y, 134 documents; and Zhang L, 134 documents.

**Fig. 2:**
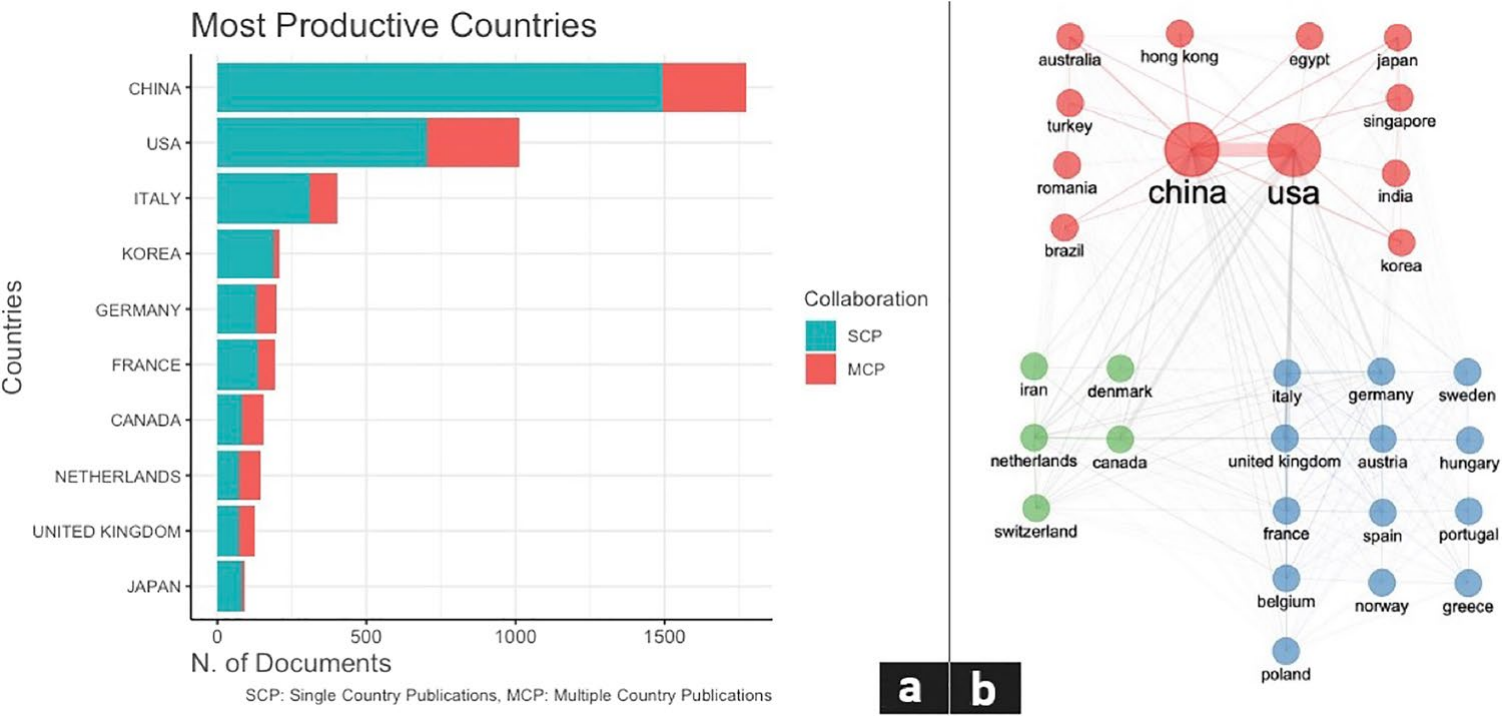
**a** Most productive countries chart, divided by single country publications (SCPs) and multiple country publications (MCPs). **b** Map of the clustered collaboration network analysis of the top 30 most productive countries

An insight of the Corresponding Authors’ Countries has been performed, followed by a subdivision of single and multiple country publications. China has reached 1773 documents published, with a Single Country Publication ratio (SCPr) of 84.2%, followed by USA with 1015 documents and SCPr of 69%, Italy with 400 documents and SCPr of 77%, Korea with 208 documents and SCPr 91.3%, and Germany with 196 documents published and SCPr of 66.3%. Qualitatively, it is possible to notice that European countries, including the most productive ones, fall within the same cluster, with the sole exception of the Netherlands (Fig. 2a and b).

Fudan University, China, is the most relevant authors’ affiliation based on number published articles (*n* = 292), followed by Memorial Sloan Kettering Cancer Center, USA (*n* = 230) and Sichuan University, China (*n* = 204). A full list of the top 10 most relevant affiliations is available in Table 1, with the Fudan University ranking as first thanks to a total number of 292 published articles.

**Table 1:** List of the top 10 most relevant authors’ affiliations

Affiliation	Articles
Fudan University, China	292
Memorial Sloan Kettering Cancer Center, USA	230
Sichuan University, China	204
Institute Of Automation, China	190
University Of Pennsylvania, USA	189
Southern Medical University, China	186
University Of Toronto, Canada	178
Capital Medical University, China	161
Harvard Medical School, USA	160
University of Chinese Academy of Sciences, China	150

A collaboration network analysis of the top 30 most productive countries has been performed, using Louvain clustering algorithm with normalization based on association. Isolated nodes have been removed and a minimum number of edges of 1 has been considered. We identified 3 main clusters of collaboration, the first one involving China and USA as most productive players, the second with Italy and Germany as most productive players, and the last one with Canada and the Netherlands (Fig. 2b).

**Fig. 3:**
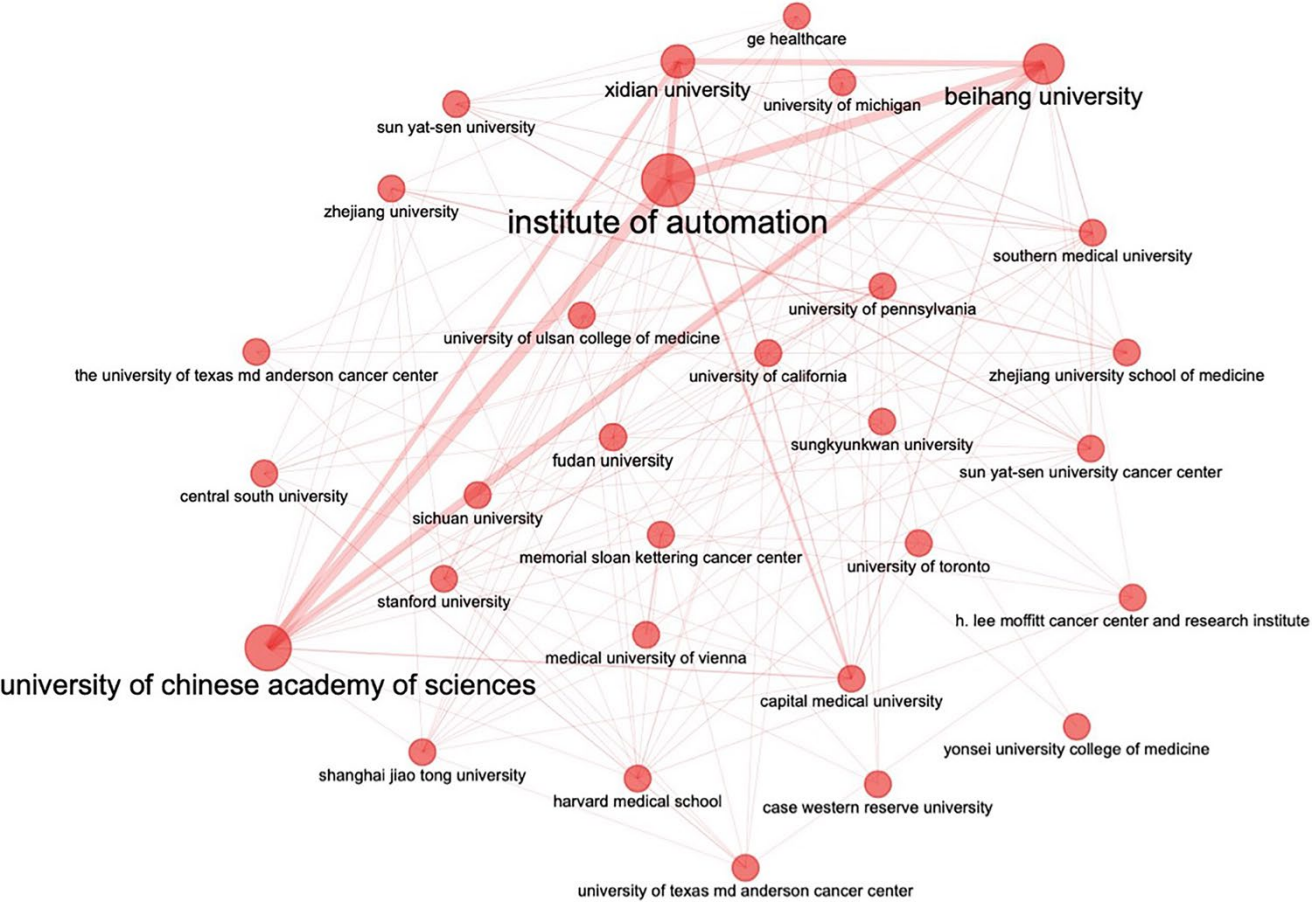
Map of the unclustered collaboration network analysis of the top 30 most productive authors’ affiliations

A further analysis of interplaying institutions collaboration network has been performed with no clusterization method applied (Fig. 3).

### Citations

As showed in Table 2, the 10 most global cited documents have been identified.

**Table 2:** List of the 10 most global cited documents. *DOI*, digital object identifier

Paper	Title	DOI	Total citations	Citations per year
GILLIES RJ, 2016, RADIOLOGY	Radiomics: Images Are More than Pictures, They Are Data	10.1148/radiol.2015151169	3024	432.00
AERTS HJWL, 2014, NAT COMMUN-a	Decoding tumor phenotype by noninvasive imaging using a quantitative radiomics approach	10.1038/ncomms5006	2465	273.89
LAMBIN P, 2012, EUR J CANCER	Radiomics: Extracting more information from medical images using advanced feature analysis	10.1016/j.ejca.2011.11.036	2209	200.82
VAN GRIETHUYSEN JJM, 2017, CANCER RES	Computational Radiomics System to Decode the Radiographic Phenotype	10.1158/0008–5472.CAN-17–0339	1497	249.50
LAMBIN P, 2017, NAT REV CLIN ONCOL	Radiomics: the bridge between medical imaging and personalized medicine	10.1038/nrclinonc.2017.141	1484	247.33
KUMAR V, 2012, MAGN RESON IMAGING	Radiomics: the process and the challenges	10.1016/j.mri.2012.06.010	1126	102.36
BAKAS S, 2017, SCI DATA	Advancing The Cancer Genome Atlas glioma MRI collections with expert segmentation labels and radiomic features	10.1038/sdata.2017.117	855	142.50
HUANG YQ, 2016, J CLIN ONCOL	Development and Validation of a Radiomics Nomogram for Preoperative Prediction of Lymph Node Metastasis in Colorectal Cancer	10.1200/JCO.2015.65.9128	833	119.00
ZWANENBURG A, 2020, RADIOLOGY	The Image Biomarker Standardization Initiative: Standardized Quantitative Radiomics for High-Throughput Image-based Phenotyping	10.1148/radiol.2020191145	606	202.00
PARMAR C, 2015, SCI REP	Machine Learning methods for Quantitative Radiomic Biomarkers	10.1038/srep13087	541	67.63

The text analysis of the titles of the top 10 cited documents has not showed any significant frequent word trend, except for “Radiomics/Radiomic” and “quantitative”.

### Keywords, keyword co-occurrences

**Fig. 4:**
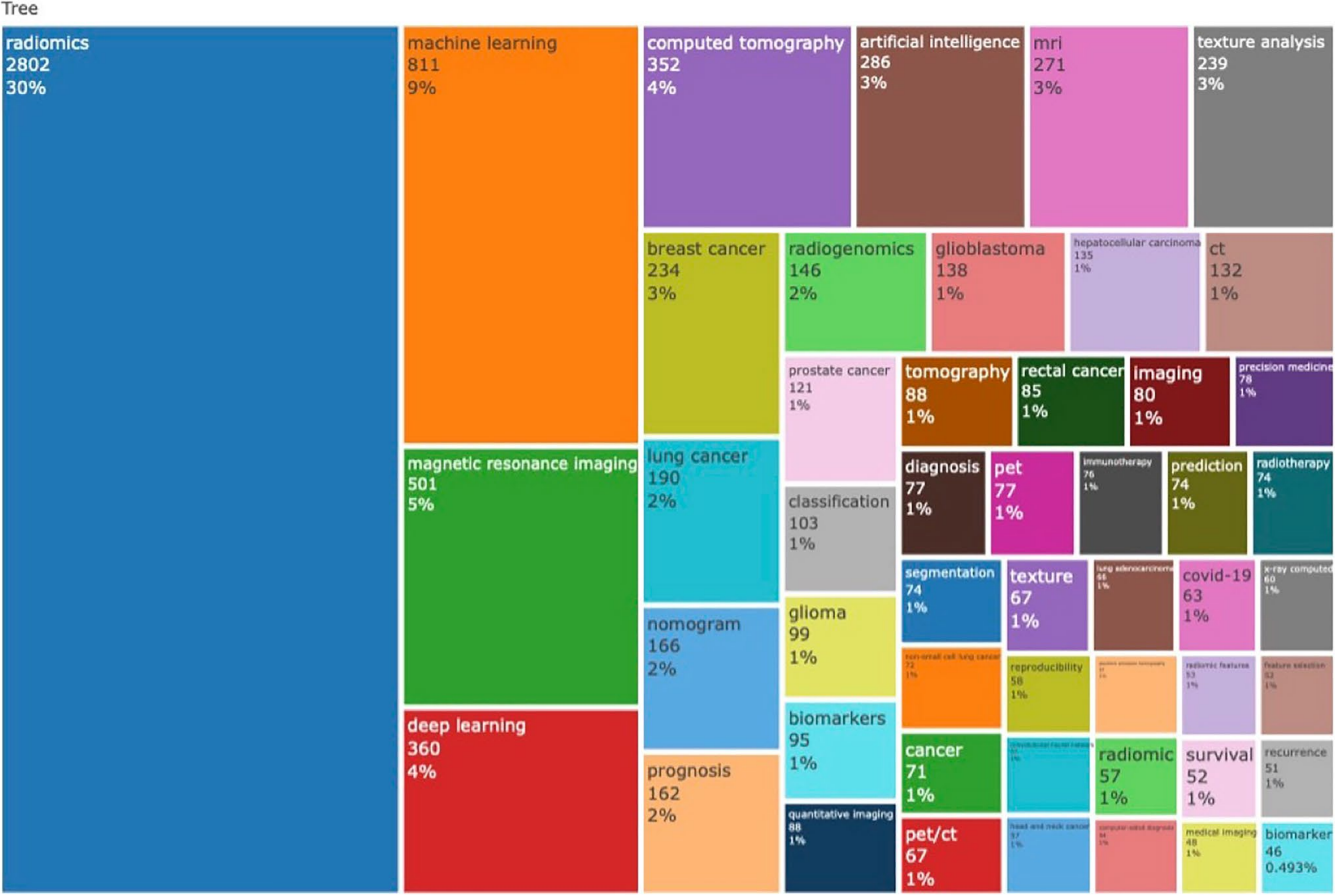
Treemap of the 50 most frequent author keywords

The articles’ author keywords were analyzed to assess the different topics underlying the Radiomics macro-area. The 50 most frequent author keywords have been plotted in the TreeMap in Fig. 4.

**Fig. 5:**
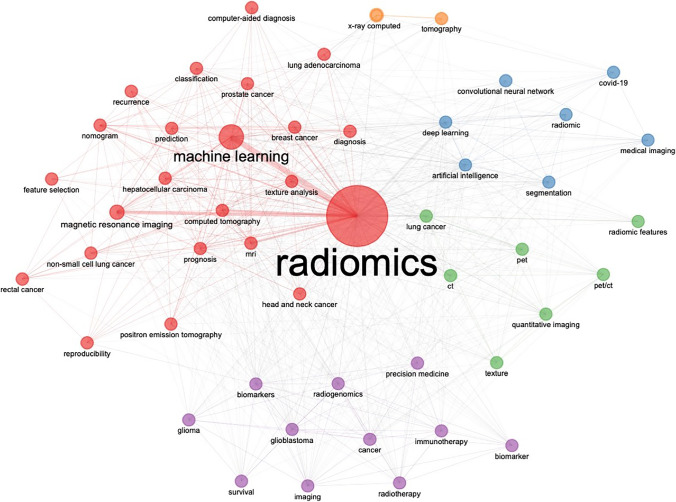
Map of the clustered co-occurrence network analysis based on top 50 authors’ keywords

We have executed a co-occurrence network analysis based on top 50 authors’ keywords and applied Louvain clustering algorithm, removing isolated nodes, and setting the minimum number of edges at 2. Five words clusters have been identified (namely, Radiomics, computed tomography, radiogenomics, deep learning, tomography), as shown in Fig. 5.

**Fig. 6:**
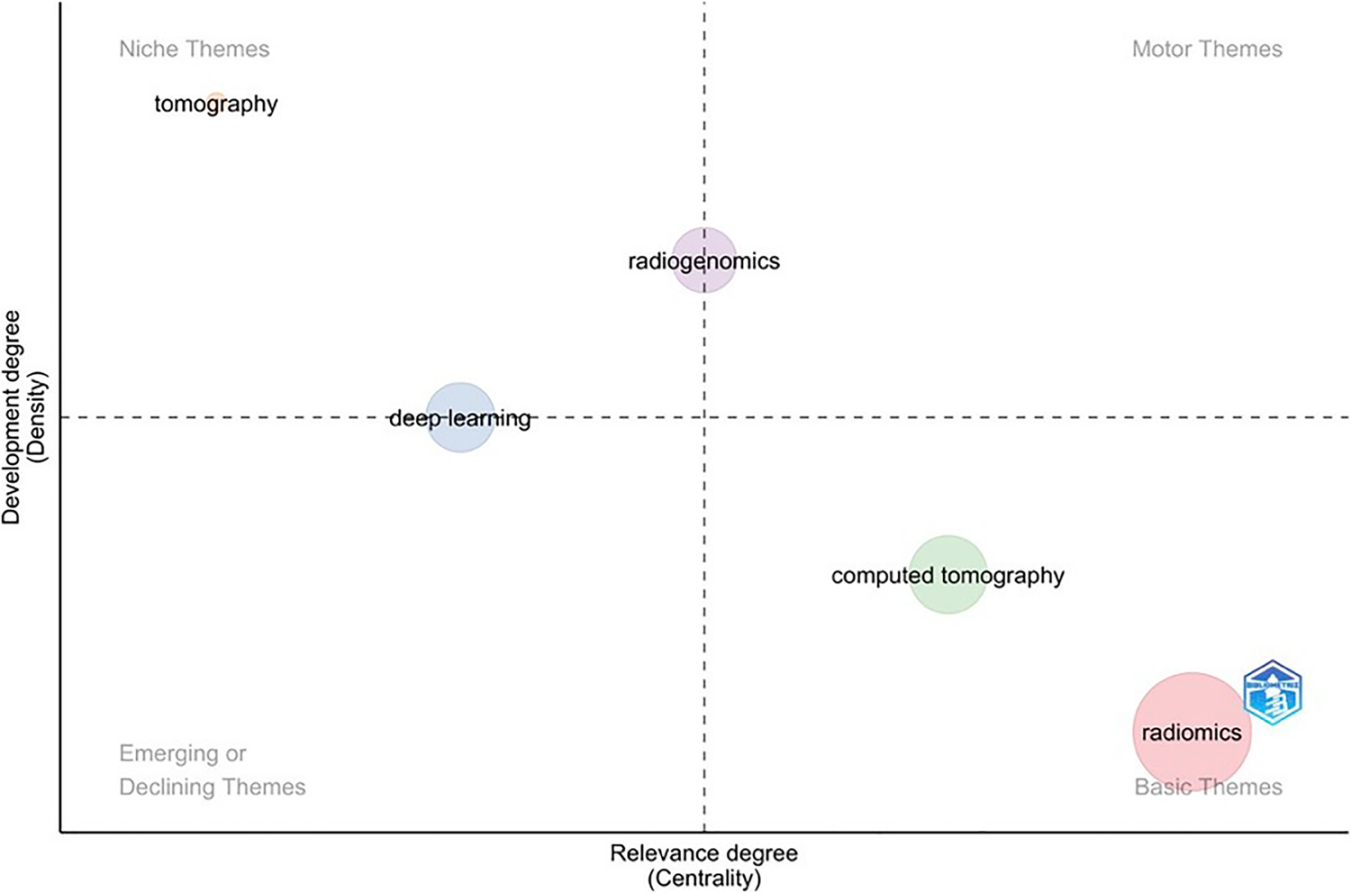
Thematic map analysis of the five identified clusters

Thematic map analysis [16] based on the five identified clusters has been performed showing their development degree (density) and the relevance degree (centrality) in Fig. 6. This strategic diagram has allowed the identification of the hot topics (higher values of centrality and density) in the upper-right quadrant, the basic topics (higher values of centrality and lower values of density) in the lower-right quadran, the peripheral topics (lower values of centrality and density) in the lower-left quadrant, and the niche topics (lower values of centrality and higher values of density). The last two represent respectively the topics which have not been fully developed and the topics which have been strongly developed but still have a marginal position in the domain under investigation.

The trend topics analysis, based on keywords frequency, for year 2021 further showed an increased interest in artificial intelligence (*n* = 286), nomogram (*n* = 166), hepatocellular carcinoma (*n* = 125), COVID-19 (*n* = 63), and X-ray computed (*n* = 60).

## Discussion

Our study shows the state of art of the current literature in Radiomics by using a standardized and easily reproducible ML methodology. In 10 years since the publication of the first article about Radiomics, the improvement in data imaging technology has supported the increasing interest in Radiomics, heading to a quantitative approach demand in the image analysis path. Further developments, such as commercial and open-source software implementing artificial intelligence, have rocketed the scientific production in the last 5 years, leading to the consolidation of the computational mean of analysis in medical imaging. The fast-growing number of documents is well indicated by the 10-year annual percentage growth rate of almost 120%.

The fast-growing interest in the topic has led Radiomics not to achieve a standardized methodology of analysis, which could vary among different authors and articles. This is reflected by the relative lack of consolidation in current literature, which is characterized by a consistent presence of articles (*n* = 3829) and conference papers (*n* = 604), with only a scarce representation of books (*n* = 2) and books-chapter (*n* = 45). A trend intent of consolidation could be showed by the increasing number of reviews (*n* = 758) and by the desumed ratio between original articles and reviews (R = 5.05). Admittedly, the ML approach implemented here has not allowed further analysis on the nature (either narrative or systematic), of the included reviews, which have further addressed this issue. Additionally, in Scopus it is not possible to address metanalyses as a distinct article category, being them classified as part of reviews.

Considering international collaborations, our data show a considerable prevalence of SCPs among the top five most productive countries, with percentages ranging from 66.3 to 91.3% for Germany and Korea, respectively. Of note, MCPs have been more common in Canada, the Netherlands, and in the UK, with a prevalence approximating the one of SCPs.

From a qualitative standpoint, it is also possible to notice the existence of a broader cluster of European countries, albeit with varying degrees of edges between individual nodes. In this regard, it is worth noticing that—to date—few European-level initiatives exist to foster international collaborations on Radiomics. Of these, the EuCanImage—funded by the European Union’s Horizon 2020 research and innovation program—is a promising example of academic-industrial-clinical partnership of 20 institutions, with the aim of realizing integrative decision support systems for precision oncology through optimized data sharing, Radiomics, and artificial intelligence applications [17]. A comparable effort is being sponsored by European funds to promote cross-border collaboration, under the name of Euradiomics [18]. Collectively, these initiatives may represent a concrete step forward in the field, thanks to the possibility of collecting large amounts of imaging and clinical metadata, to encourage collaboration across participating institutions and to promote the development of advanced algorithms under the principles of federated learning, while promoting the real-life clinical application of this emerging discipline [19, 20].

The collaboration network analysis of the top 30 most productive countries provides further insight into these data, indicating that the most relevant cluster of collaboration occurs between China and USA, followed by two smaller clusters constituted by Italy and Germany, and Canada and the Netherlands as the second and third most productive players, respectively.

It is widely known that Radiomics needs specific skills to ensure an adequate and reliable its pipeline. Furthermore, multidisciplinary competences can help critical reasoning in achieving correct conclusions from data analysis. Therefore, the need of dedicated teams should be considered crucial for an effective scientific production. This scenario is well represented in Table 1, which shows the unclustered data analysis of the main institutions contributing to document production. Nowadays, most of the literature is limited to a small number of institutions, arguably having high specialization in the field, and probably relying on well-established cooperative networks. The three most relevant affiliations for number of articles produced are “Fudan University” (*n* = 292), “Memorial Sloan Kettering Cancer Center” (*n* = 230), and “Sichuan University” (*n* = 204). No European institutions are present in the top 10 more relevant affiliations ranking.

An analysis of the most cited documents has been performed to ascertain the interest and nature of the articles which have had major impact in the scientific literature of the field (Table 2). The first three most cited articles are milestones in the Radiomics research and account for 7698 citations in total, which is the 7.97% of the total number of overall citations of the papers included in our analysis. The first most cited article [21] was written in 2016 by Gillies et al. and introduces the new paradigm of radiological images as valuable data that could be mined and classified by semi-automatic methods to accommodate patients’ features even beyond imaging, representing a promising tool for the diagnosis and prognosis of malignancies. Among the other topics, the challenges of reproducibility, of the big data analysis and the data sharing are discussed, as well. The second most cited article [22] was written in 2014 by Aerts et al. and focuses on the usage of the quantitative Radiomics approach for defining a prognostic value of a Radiomic signature in non-small cell lung cancer. This study tightly associates the usage of the Radiomic data with the clinical-world ones, showing the feasibility of a pipeline which includes a features unsupervised clustering technique. The third most cited article was written in 2012 [1] by Lambin et al. and it is not only the first written article on Radiomics, but it also introduces the concept of quantitative analysis of medical imaging data through automatic or semi-automatic software that can provide more and better information than those inferred by physicians. Among the 10 most cited documents, it is of valuable interest the presence of the article “The Image Biomarker Standardization Initiative: Standardized Quantitative Radiomics for High-Throughput Image-based Phenotyping” [23], published in 2020 by Zwanenburg et al., and is one of the first initiatives in the Radiomic field for the standardization of features extraction and analysis, mirroring the growing sensibility towards the topic of reproducibility in Radiomics studies. Furthermore, the latter study could be broadly seen as a bastion of the need of effective validation of radiomic signatures in the clinical practice, which is advisable to be concentrated on specific pathologies and clinical outcomes that have demonstrated the beneficial role of the clinical impact of radiomics.

Considering the keywords, we have analyzed the absolute top 50 author-keywords, dividing them in functional semantic groups (Fig. 5). The first group of words, which is the most represented in the document population, includes the methodological insight of the field, with keywords such as “machine learning”, “magnetic resonance imaging”, “deep learning”, “computed tomography”, “artificial intelligence”, “quantitative imaging”. The second group includes word that are close to the prognostic value of Radiomics, such as “nomogram”, “prognosis”, “radiogenomics”, “classification”, “biomarkers”, “diagnosis”. The third group includes the pathologies in which Radiomics has been performed, such as “breast cancer”, “lung cancer”, “prostate cancer”, “glioma”, “glioblastoma”. In the current analysis, many words have been repeated different times, using acronyms or substantive/adjective forms (e.g., “Radiomics” and “Radiomic”, “magnetic resonance imaging” and “mri”, etc.), showing a deep fragmentation of the keyword panorama. The present keyword heterogenicity could be probably due to the emerging nature of the field, but further consolidation may be needed to optimize the search strategy for system reviewing approach.

The adopted ML approach has showed five thematic co-occurrence clusters, mirroring the most common associations in the interdisciplinary common ground. The first cluster involves the term “Radiomics” tightly bound to “machine learning”, involving different keywords regarding some cancer pathologies and their prognosis (hepatocellular carcinoma, lung adenocarcinoma, breast cancer, nomogram, prediction) and some others regarding technical aspects (feature selection, classification, reproducibility). The presence of “nomogram” in the 2% of the total number of author keywords probably reflects the intent of providing clinicians with user-friendly tools, for a more immediate integration of Radiomics-derived information into routine pathways of care. This cluster in the thematic map analysis is both a basic and motor theme, in which most of the scientific articles are so far concentrated and in which it is our fervent aspiration that, through further consolidated efforts, more studies would ensure the respect of the impact of radiomics in the current clinical practice in the near future. The second cluster involves the terms “radiogenomics” and “precision medicine”, interconnecting with “immunotherapy”, “biomarker” and brain tumors, showing multi-omics application as emerging field in the current literature. The third cluster found its root in the machinery basin, involving terms as “PET”, “CT” and “Quantitative imaging”, covering a central role as basic and motor theme. The fourth cluster deals with the “artificial intelligence” and “deep learning”, including “convolutional neural network” and “COVID-19”, covering a central position as emerging theme, but still limited to a small number of documents in comparison to other fields of scientific production. It is interesting to notice that—as of the 31st of December 2021—63 publications had “COVID-19” among the author keywords, thus ranking among the top 50 keywords in Radiomics. Considering the relatively small timeframe in which COVID-19 became of interest in the longer lifespan of Radiomics, it is straightforward to observe that researchers worldwide have promptly tried to exploit the potentials of Radiomics from the very earliest phases of the pandemic outbreak. As last cluster, we found the co-occurrence of “X-ray computer” and “tomography”, which has many inbound and outbound connections with the Radiomic cluster and covers a supportive role for the keywords inside the methodology area.

We could perform a complete set of ML-based bibliometric analyses, thus providing an unbiased overview of the state of art of Radiomics in its first 10 years of life. Several aspects of the publication landscape that would otherwise be left unnoticed at human inspection, could be highlighted, and commented, including, but not limited to, type of publications, SCPs/MCPs pattern, institutions collaboration, and author keywords networks. In this sense, our work can be considered an integration to a previous effort in the field by Ding et al. [24]. In their work, the authors have realized the first bibliometric analysis of Radiomics publications using CiteSpace, a “generic approach to detecting and visualizing emerging trends and transient patterns in scientific literature” [25]. However, Ding et al. have restricted their search to the sole field of oncology and have decided to exclude all publications other than full-texts and reviews, which somehow limits the field of analysis and excludes potentially relevant topics, such as COVID-19. Moreover, we believe that the inclusion of all publication types (e.g., books, chapters, and conference abstracts) can be informative in delivering a complete overview on how knowledge on a specific topic can be spread out of the more common track of indexed journals, which may be affected by long publication times, need of subscription, and other factors.

Admittedly, our work still presents some limitations. Firstly, the dataset was retrieved from a single electronic database (Scopus), which may have at least partially affected our findings. Secondly, it was not possible to perform a reliable analysis of neither citation trends or co-citation networks, as we could not account for citations deriving from works deriving from other sources and/or not included in our search. Finally, the number of citations changes over time, so this part of the analysis should be considered provisional, and prone to modification in the upcoming months/years.

## Conclusions

Our work demonstrates the importance of bibliometric analysis to detect unknown pattern of data in Radiomics publications. Specifically, we captured a raising interest in the field, the most relevant collaborations, keywords co-occurrence network and trending topics. However, some pitfalls still exist, including the scarce standardization, the relative lack of homogeneity across studies, and the absence of real-life applications in the clinical practice. To overcome this issue, we suggest an optimization of keywords and the creation of a common ground knowledge, promoting a deeper network of MCPs, while ensuring more solid reproducible pipelines which can be easily translated in clinical practice.

## Supplementary Information

Below is the link to the electronic supplementary material.

Supplementary file1 (PDF 131 KB)

## Declarations

### Guarantor

The scientific guarantor of this publication is Federico Mastroleo.

### Statistics and biometry

One of the authors has significant statistical expertise.

### Methodology

• Multicentre study
