# Supplementary figures and images for: Quo vadis Radiomics? Bibliometric analysis of 10-year Radiomics journey
Source: Eur Radiol. 2023 Apr 18;33(10):6736–45. doi: 10.1007/s00330-023-09645-6 (PMC10110486; doi:10.1007/s00330-023-09645-6)

Suppl. Mat. Fig. 1: Workflow diagram of the bibliometric analysis

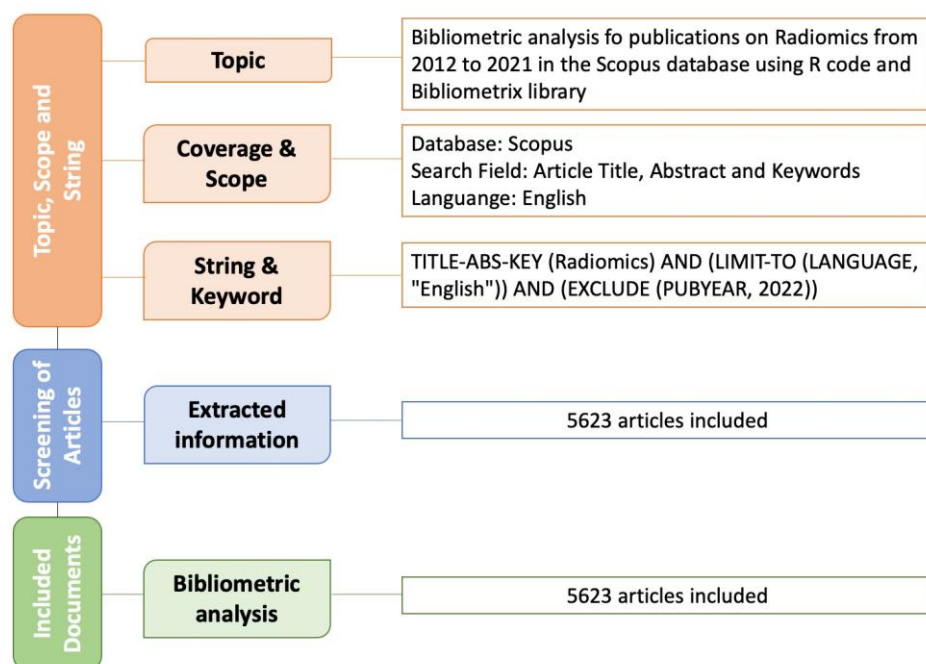

Supplement: Supplementary file 1 — Supplementary file1 (PDF 131 KB) [file 330_2023_9645_MOESM1_ESM.pdf]
